# Roles of exosomes in immune regulation of osteoarthritis and their applications in inflammation repair

**DOI:** 10.3389/fimmu.2025.1611718

**Published:** 2025-09-04

**Authors:** Shuquan Lan, Chao Zhang

**Affiliations:** Stomatological Hospital, School of Stomatology, Southern Medical University, Guangzhou, China

**Keywords:** osteoarthritis, immune regulation, inflammation repair, synovial inflammation, exosomes, mesenchymal stem cells

## Abstract

Osteoarthritis (OA) is a chronic, degenerative joint disease characterized by progressive cartilage degradation and inflammation. Exosomes, small vesicles released by various cell types, play a crucial role in mediating immune responses and inflammation. In OA, exosomes from antigen-presenting cells (APCs) promote synovial inflammation through antigen presentation and cytokine signaling, while those from mesenchymal stem cells (MSCs) modulate inflammation by reprogramming macrophages. Exosomal cargo has shown potential in controlling inflammatory pathways and protecting cartilage from degradation. MSC-derived exosomes have demonstrated therapeutic promise in reducing OA-related inflammation and promoting cartilage regeneration. Despite several reports have outlined the role of exosomes or immune modulation in OA individually, comprehensive reviews integrating their roles in both immune regulation and inflammation repair in OA are still lacking. This knowledge gap hinders the translational application of exosome-based interventions in clinical settings. This review aims to summarize the immunoregulatory roles of exosomes in OA, emphasizing their impact on inflammation and immune responses, and discusses their therapeutic potential in OA treatment. By elucidating the roles of exosomes, the findings of this review could facilitate the development of novel, minimally invasive strategies for improving OA treatment and enhancing inflammation repair.

## 1 Introduction

Osteoarthritis (OA) is a prevalent, chronic, age-related joint disease characterized by progressive cartilage degeneration, synovial inflammation, and subchondral bone remodeling (1, 2). Traditional therapies for OA are primarily palliative, focusing on symptom relief rather than disease modification. Nonsteroidal anti-inflammatory drugs (NSAIDs) and corticosteroids alleviate pain but are associated with significant adverse effects upon long-term use (3, 4). Intra-articular injections of corticosteroids or hyaluronic acid offer only transient relief, while physical therapy maintains mobility without halting cartilage loss (5, 6). Surgical interventions such as joint arthroplasty are effective in advanced cases but are invasive and costly (7).

In contrast, exosome-based therapies have emerged as a promising regenerative strategy, targeting the immunological and catabolic pathways central to OA pathogenesis (8, 9). MSC-derived exosomes can modulate the inflammatory microenvironment by promoting M2 macrophage polarization and inhibiting NF-κB signaling, thereby promoting cartilage repair (10, 11). These nano-vesicles can also deliver specific miRNAs to restore chondrocyte homeostasis and extracellular matrix synthesis. Although currently in preclinical or early clinical stages (12–14), exosome-based approaches offer minimally invasive, repeatable, and potentially disease-modifying treatment options. Existing literature has predominantly examined either the effects of exosomes or immune regulation in OA separately (15–17), with seldom systematic evaluation of their combined impact on both inflammatory suppression and structural repair. This lack of integrated understanding poses a barrier to the clinical translation of exosome-based therapies. This review aims to highlight recent advances in understanding the immunoregulatory functions of exosomes in OA and their therapeutic implications in inflammation resolution and cartilage regeneration, providing a timely and in-depth evaluation of exosome-based interventions for inflammation resolution and cartilage regeneration in OA.

## 2 Properties of exosomes

### 2.1 Structural features

Although exosome molecular composition varies with the cell of origin, microenvironment, developmental stage, epigenetic landscape, and precise biogenetic pathway, exosomes share several conserved features. An exosome comprises an external “shell” and an internal “cargo.” The limiting membrane displays a lipid-raft-like architecture enriched in cholesterol, sphingomyelin, and ceramide, which facilitates vesicular trafficking within the cytosol (18). Canonical surface markers include heat-shock proteins, tetraspanins (CD82, CD81, CD63, and CD9), and major histocompatibility complex (MHC) molecules (19). TSG101, which binds ubiquitinated cargo proteins, serves as a hallmark of endosomal sorting. Initially regarded as metabolic waste, exosome contents are now known to encompass abundant nucleic acids, lipids, and proteins, including long non-coding RNA, microRNA, and mRNA, that play critical roles in intercellular communication and immune regulation.

### 2.2 Biogenesis and release

Exosome biogenesis and secretion constitute a tightly regulated, multi-step process initiated by the inward budding of the plasma membrane to form early endosomes. These compartments subsequently mature into multivesicular bodies (MVBs) through a secondary invagination process that requires coordinated action of key molecular machinery, including the endosomal sorting complex required for transport (ESCRT), particularly ESCRT-III, which mediates vesicle budding and scission (20). While ESCRT-dependent mechanisms predominate in most cell types, alternative ESCRT-independent pathways involving sphingomyelinase-mediated ceramide generation can also facilitate exosome formation. Following their biogenesis, exosomes are secreted through three primary mechanisms: (i) fusion of MVBs packed with exosomes to the plasma membrane; (ii) direct budding outward from the plasma membrane; (iii) discharge from intracellular plasma-membrane-connected compartments (IPMCs) after relief of export restrictions (21, 22). Sustained mTORC1 activation inhibits exosome secretion, while mTORC1 blockade promotes it, with both processes being linked to autophagy (23).

### 2.3 Exosomes and immunomodulation

In 1996, immunologists first observed that B lymphocytes transformed by the Epstein-Barr virus could produce exosomes via fusion between MVBs and the plasma membrane (24). Subsequent studies revealed that numerous immune and non-immune cells, including T cells, B cells, dendritic cells (DCs), and macrophages, release exosomes capable of mediating immune activation or suppression (25). Exosomal immunoactivity affects both innate and adaptive immunity by modulating antigen presentation, T cell activation, regulatory T cell polarization, immunosuppression, and anti-inflammatory pathways. Exosomes play a vital role in activating and enhancing immune responses via antigen presentation. Professional antigen-presenting cells, including macrophages, B cells, and dendritic cells, release exosomes containing abundant costimulatory signals and MHC class I/II molecules. These exosome-associated peptide antigens are essential for regulating immune function (26).

## 3 Synovial inflammation and immune dysregulation in OA

OA is a complex degenerative joint disease involving multiple pathological factors. It progressively destroys articular cartilage, leading to persistent pain and gradual loss of joint function (27). Emerging evidence indicates that low-grade synovial inflammation plays a crucial role in both the initiation and advancement of osteoarthritis (28). The healthy synovium comprises two distinct layers: an outer vascular (sub-intimal) layer and an inner cellular (intimal) layer. Together, these layers secrete synovial fluid, thereby minimizing the coefficient of friction across the articular surface (29). The sub-intimal layer is comparatively thick and consists of adipose tissue interspersed with lymphatic vessels, nerve fibers, dense fibrous tissue, type-I collagen, and microvasculature. The intimal layer is thinner and populated by synovial fibroblasts and synovial macrophages (30). During OA development, the synovium undergoes intimal hyperplasia, stromal fibrosis, and neovascularization, accompanied by marked infiltration of NK cells, plasma cells, B cells, mast cells, T cells, and macrophages (31). Notably, macrophage infiltration is already evident at the earliest stages of disease (32). This inflammatory cascade is initiated by localized chondrocyte injury, increased vascularization, and damage-associated molecular pattern (DAMP) release, which collectively activate synovial macrophages and lymphocytes. The resulting immunocyte activation triggers a feed-forward inflammatory loop characterized by elevated chemokine and cytokine production (33). Concomitantly, dysregulated chondrocytes secrete matrix metalloproteinases, pro-inflammatory cytokines, and prostaglandins, thereby creating a self-perpetuating cycle of cartilage destruction (34).

## 4 Exosomes in the immunoregulation of OA

Both exosomes and OA are intimately linked to immune homeostasis; exploiting this nexus offers new therapeutic avenues (35). Following uptake by target cells, distinct exosome populations elicit discrete functional outcomes. Exosomes may interact directly with the immune system by presenting antigens or, alternatively, modulate cellular behavior via cargo microRNAs (miRNAs) (36). Moreover, exosomes can fuse with endosomes within recipient cells, undergoing self-degradation or being re-secreted extracellularly.

### 4.1 APC-derived exosomes and OA

#### 4.1.1 Lymphocyte-derived exosomes

Upon T-cell-receptor engagement, murine CD4^+^CD25^+^Foxp3^+^ Tregs release exosomes (37). Treg-derived vesicles carrying miRNAs suppress T helper cell type 1 (Th1) responses via non-cell-autonomous gene silencing (38). Activated CD8^+^CD25^+^Foxp3^+^ T cells likewise secrete exosomes that inhibit CD8^+^ cytotoxic T-lymphocyte activity, representing an intrinsic negative-feedback mechanism to forestall excessive inflammation. In OA, this pathway manifests as the slow evolution of synovitis: despite strong activation signals from APC-derived exosomes, T-cell hyper-responsiveness is restrained, preventing precipitous inflammatory escalation (39). Compared to DC-derived exosomes, B cell-derived exosomes have been less extensively studied. B-cell exosomes are detectable very early after antigenic challenge—even earlier than DC exosomes—and can activate APCs. These vesicles are enriched in B7-1/B7-2, MHC class I and II molecules, and intercellular adhesion molecule 1 (ICAM-1), facilitating CD4^+^ T-cell activation and antigen presentation (40). In early OA, B cell-derived exosomes contribute to synovitis. Functional studies show that integrins expressed on these vesicles mediate adhesion to extracellular matrix components and cytokine-primed fibroblasts, suggesting a novel long-distance conduit for adhesive signaling during inflammation. B-cell exosomes also enhance C3 deposition and T-cell reactivity, thereby intensifying synovitis and fueling OA progression (41).

#### 4.1.2 Dendritic cell-derived exosomes

Investigations into exosomal immunomodulation were initiated with DC exosomes, which are now well characterized. Exosomes from mature DC**s** display MHC class II molecules and co-stimulatory ligands such as B7-2 and ICAM-1, enabling direct T-cell activation (42). In contrast to their mature counterparts, immature DC-derived exosomes exhibit distinct immunomodulatory properties. Rather than directly activating T cells, these vesicles primarily facilitate antigen distribution to other antigen-presenting cells (43, 44) or mediate the transfer of MHC/antigen complexes to DC surface receptors, thereby indirectly promoting CD8^+^ T cell polarization (45). Mechanistically, immature DC exosomes are characterized by reduced expression of co-stimulatory and adhesion molecules, while displaying up-regulated immunosuppressive factors (TGF-β, NKG2D ligands, Galectin-9) and CD95L, features that collectively induce T cell apoptosis and suppress immune activation (46, 47). Within the osteoarthritic joint, while both DC subsets contribute to T cell-mediated inflammation, immature DC exosomes demonstrate a paradoxical protective capacity by attenuating synovial inflammatory cell infiltration and potentially slowing cartilage degeneration (48).

#### 4.1.3 Monocyte macrophage-derived exosomes

**Figure 1:**
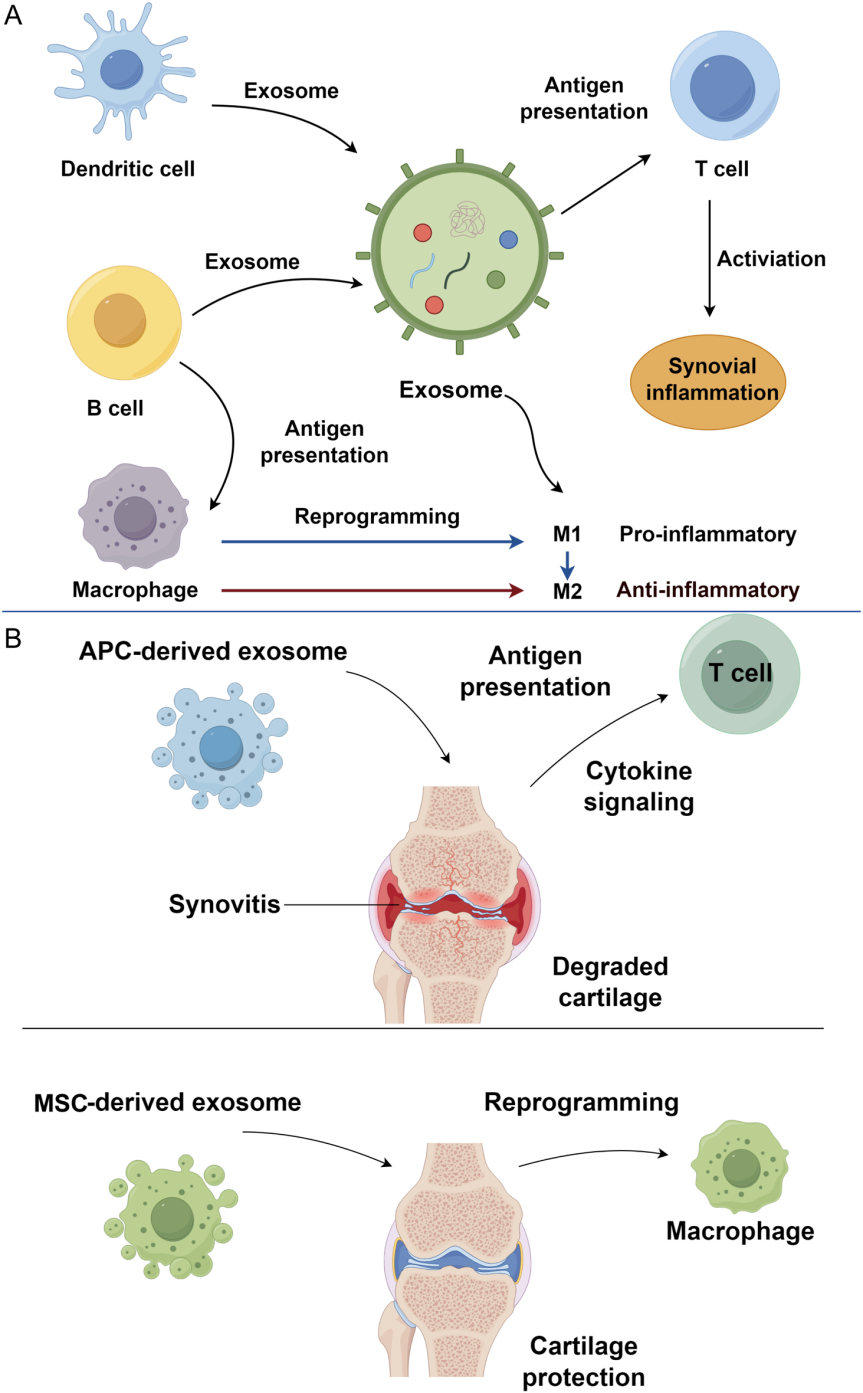
Immune cell-derived exosomes in osteoarthritis progression. **(A)** Exosomes derived from immune cells, including dendritic cells, B cells, and macrophages, mediate immune communication and synovial inflammation in osteoarthritis. **(B)** APC-derived exosomes contribute to osteoarthritis progression. In contrast, MSC-derived exosomes exert therapeutic effects by reprogramming macrophages toward an anti-inflammatory phenotype, thereby protecting cartilage and alleviating inflammation. APC, antigen-presenting cell; MSC, mesenchymal stem cell.

Macrophage-derived exosomes play a pivotal role in propagating inflammation in osteoarthritis through their immunomodulatory effects on innate and adaptive immune cells. Following bacterial infection, these exosomes exert pro-inflammatory effects by activating naïve macrophages, promoting dendritic cell maturation, and stimulating CD4^+^ and CD8^+^ T cell responses. This occurs via the presentation of bacterial antigens, such as immunogenic proteins, which trigger DC activation and subsequent cytokine release (49). Although initially characterized in infectious contexts, such antigen presentation constitutes a broader mode of inter-immune-cell communication. In OA, persistent cartilage damage and osteophyte formation sustain a chronic inflammatory microenvironment, where macrophage-derived exosomes contribute significantly. Exosomes isolated from OA synovial fluid polarize macrophages toward an M1 phenotype, driving the production of pro-inflammatory mediators, including chemokines (CCL20, CCL15, CXCL1), cytokines, and matrix metalloproteinases (MMP-12, MMP-7) (50, 51) (**Figure 1A**).

### 4.2 Exosomal cargos in osteoarthritis pathogenesis

To contextualize the roles of exosomal cargos in OA pathogenesis, it is essential to examine how epigenetic alterations mediated by these molecules drive disease progression. The molecular payload enclosed within exosomes is intimately involved in the immunomodulation of osteoarthritis. Throughout disease onset and progression, epigenetic alterations, including miRNA repression, histone modification and DNA methylation (52), perturb multiple transcriptional programs and the synthesis of proteolytic factors that govern the anabolic–catabolic equilibrium, such as ADAMTS5, MMP-13 and RUNX2. Among these epigenetic regulators, miRNAs have attracted particular attention in OA pathobiology (53).

Within the joint, miRNAs exert their effects by targeting key mediators of cartilage homeostasis. Current evidence shows that miR-140 is down-regulated in osteoarthritis cartilage and in chondrocytes stimulated with IL-1β, underscoring its chondroprotective function (54). Mice lacking miR-140 exhibit proteoglycan loss and cartilage fibrillation, whereas transgenic over-expression confers resistance to experimental arthritis (55). This regulatory role is further emphasized by the inverse relationship between miR-140 and catabolic enzymes. In osteoarthritis tissues, low level of miR-140 expression is typically accompanied by increased MMP-13 and ADAMTS5, thereby accelerating disease progression (56, 57). Mechanistically, miR-140 exerts its protective effects primarily by directly targeting the 3′-UTR of ADAMTS5 and MMP-13 mRNA, suppressing their translation and thus attenuating matrix degradation (58). Furthermore, miR-140 negatively regulates RALA and SMAD3, which concurrently modulates chondrocyte hypertrophy and reduces catabolic gene expression (59). Exosomal delivery of miR−140 to chondrocytes enhances COL2A1 expression while simultaneously inhibiting IL−1β-induced activation of the NF-κB pathway, leading to reduced synthesis of pro-inflammatory mediators such as inducible nitric oxide synthase (iNOS) and cyclo−oxygenase−2 (COX-2) (60, 61). Moreover, miR−140-containing exosomes prevent oxidative stress-induced apoptosis by targeting P38 MAPK signaling in articular cartilage (62). Together, these multifaceted mechanisms underscore the central role of exosomal miR−140 in restoring the anabolic-catabolic balance within the joint microenvironment and protecting against OA-associated cartilage erosion. Additional miRNAs, including miR-139 and miR-9, similarly disrupt the anabolic–catabolic balance, precipitating extracellular-matrix breakdown and chondrocyte injury (63).

The dysregulation of exosomal miRNAs in OA is not merely a bystander phenomenon but actively contributes to disease pathology. The miRNA composition of exosomes differs markedly between patients with OA and healthy individuals (64). miRNA profiling of synovial-fluid–derived exosomes reveal the miR-200c overexpression in OA, which suppresses ZEB1 and consequently diminishes type II collagen synthesis (65). Further evidence of exosomal miRNA dysregulation comes from inflammatory stimulation experiments: IL-1β stimulation of normal synovial fibroblasts up-regulates 340 and down-regulates 24 distinct miRNAs relative to unstimulated controls, whereas exosomes released by OA chondrocytes contain 22 up-regulated and 29 down-regulated miRNAs compared with those from normal chondrocytes (66). Collectively, these findings highlight the bidirectional relationship between exosomal cargos and OA progression. Such changes reflect both an altered joint microenvironment and the host’s immunoregulatory response; they, in turn, feed back into disease control—for example, elevated miR-140 dampens local inflammatory cytokine release and mitigates immune activation (67). Exosomes thus occupy a pivotal position in the immunological circuitry of OA.

### 4.3 Additional links between exosomes and OA

Beyond immunoregulation, exosomes intersect with several other facets of OA pathogenesis, notably synovial angiogenesis. Exosomes released by synovial fibroblasts augment vascular endothelial growth factor (VEGF) secretion, thereby stimulating angiogenesis and driving pathological progression (68, 69). Human umbilical-vein endothelial cells cultured with these exosomes display enhanced migration, tube formation and overall angiogenic capacity. Chondrocyte-derived exosomes are also implicated in osteophyte formation (68). Vesicles 20 – 200 nm in diameter, present within nascent cartilage and bone outgrowths, share exosomal features and carry mediators such as bone morphogenetic proteins (BMPs) that are indispensable for calcification and osteophyte development (70). Exosomes further influence chondrocyte metabolism. Reduced mitochondrial mass in human OA chondrocytes relative to healthy controls, whereas elevated reactive oxygen species (ROS), signifying concurrent mitochondrial dysfunction and ROS accumulation (71–73). Exosome treatment restores mitochondrial integrity, evidenced by increased intracellular ATP, while lowering ROS levels. Intra-articular administration of exosomes can therefore attenuate OA progression by rectifying chondrocyte metabolic defects (74).

## 5 MSC−derived exosomes and OA

### 5.1 Anti−inflammatory mechanisms of MSC−derived exosomes

The therapeutic potential of MSC-derived exosomes in OA hinges on their ability to reprogram the inflammatory microenvironment. MSC-derived exosomes contain bioactive components, including trophic factors and apoptosis inhibitors, that modulate the injury microenvironment by shifting the balance from pro-inflammatory to anti-inflammatory responses (75). This shift is particularly relevant in OA, where synovial macrophages play a central role in disease progression (76, 77). In OA pathogenesis, both clinical observations and experimental models demonstrate significant inflammatory cell accumulation within the synovial tissue, particularly highlighting the crucial role of synovial macrophages (78). Notably, macrophage polarization dictates their functional impact: during inflammatory processes, macrophages undergo functional polarization into two distinct subsets: the pro-inflammatory M1 phenotype and the anti-inflammatory M2 phenotype, which play counterregulatory roles in disease progression (79).

MSC-derived exosomes directly influence macrophage polarization to attenuate inflammation. Exosomes derived from MSCs, particularly those containing elevated levels of miR-135b, inhibit MAPK6 expression, which facilitates the polarization of synovial macrophages toward the M2 phenotype and subsequently reduces cartilage degeneration (80, 81). Another key mechanism involves the suppression of NF-κB signaling, a master regulator of inflammation. Targeted inhibition of NF−κB is considered a promising strategy for controlling OA−associated inflammation (82). Upon stimulation with inflammatory mediators, NF-κB translocates to the nucleus and up regulates a repertoire of inflammatory genes encoding proteins, including COX-2, MMPs, and iNOS, culminating in chondrocyte death and exacerbation of OA pathology. MSC exosomes counteract this process via specific miRNAs: phosphorylation−dependent degradation of IκB−α is a pivotal step in NF−κB activation; MSC−derived exosomes carrying miR−147b inhibit TNF−α and IL−1β- driven expression of inflammatory mediators and prevent IκB−α degradation (83, 84) (**Supplementary Table S1**).

Multiple RNAs contained in MSC exosomes modulate inflammatory signaling in OA. For example, miR−222 targets HDAC4 and thereby down−regulates MMP−13 protein (85, 86); miR−199a−5p lowers IL−6 and TNF−α levels, limiting inflammation and cartilage destruction; miR−140−5p targets Toll−like receptor 4 (TLR4), restraining proliferation of synovial fibroblasts and reducing IL−6 and IL−8 secretion, thus fostering cartilage regeneration (87, 88). Additional miRNAs contribute to the resolution of oxidative stress and inflammation. miR−9−5p down−regulates SDC1, diminishing expression of IL−1, IL−6, TNF−α, MMP−13, alkaline phosphatase (ALP), cartilage oligomeric matrix protein (COMP) and C−reactive protein (CRP), while increasing superoxide dismutase (SOD), NO, malondialdehyde (MDA), iNOS and COX−2, collectively alleviating cartilage injury and curbing inflammatory and oxidative stress damage (89, 90). Beyond miRNAs, long non-coding RNAs also play a role: the long non−coding RNA MALAT1 up−regulates miR−19b via the Wnt/β−catenin and NF−κB pathways, protecting chondrocytes from lipopolysaccharide−induced inflammatory injury (91). In addition, miR−181c, miR−146a, and miR−21 contained in MSC exosomes can reverse the pathological inflammatory milieu characteristic of OA (92, 93) (**Figure 1B**).

### 5.2 Recent advances in exosome-based OA therapy

The reparative function of MSCs and their exosomes can be modulated by diverse pre−conditioning strategies, encompassing both biomaterial−based and physical interventions. Biomaterials such as hyaluronic acid, sodium−alginate Janus microspheres and related carriers enhance MSC adhesion to cartilage and enable targeted delivery that accelerates cartilage repair (94, 95). Recent advances in exosome-based OA therapies highlight innovative strategies for cartilage repair. Preconditioning MSCs with cytokines and biomaterials, such as hyaluronic acid and Janus microspheres, enhances exosome yield and therapeutic efficacy (64, 96). Engineered exosomes, such as CRISPR/Cas9-loaded CAP-modified hybrids (CAP/FGF18-hyEXO) and fucoidan-primed exosomes (F-MSCs-Exo), promote chondrogenesis and autophagy via miR-146b-5p (97). Hydrogel encapsulation enables sustained, targeted delivery. Conversely, pathogenic FLS-derived exosomes exacerbate OA via HIF1A-driven glycolysis, which is reversible with 2-DG (98). Macrophage-derived miR-26b-5p exosomes and placental exosomes further modulate inflammation and anabolism (50). These approaches underscore exosomes’ potential in precision OA therapy through engineering, priming, and advanced delivery systems.

## 6 Conclusion

In conclusion, exosomes have emerged as pivotal mediators in the complex immunological and inflammatory processes underlying OA. These nano-sized vesicles, which are released by various cells, including antigen-presenting cells and MSCs, play dual roles in OA pathogenesis by both propagating and mitigating inflammation. APC-derived exosomes, particularly from dendritic cells, are integral in enhancing immune activation, facilitating antigen presentation, and driving synovial inflammation through cytokine signaling. These exosomes contribute to the activation of T cells and macrophages, thereby accelerating disease progression. On the other hand, exosomes derived from MSCs exhibit a counter-regulatory function, promoting anti-inflammatory responses and cartilage protection. By carrying specific microRNAs and other bioactive molecules, MSC-derived exosomes can reprogram macrophages, shifting their polarization towards the anti-inflammatory M2 phenotype, thus attenuating cartilage degradation and fostering tissue repair.

The therapeutic potential of exosomes in OA treatment is significant, particularly in their ability to influence the joint microenvironment. However, despite this promise, several challenges hinder their clinical translation. Standardization of exosome production, including isolation methods, quantification metrics, and quality control, remains unresolved, leading to batch variability and inconsistent therapeutic effects. Optimal dosing regimens, frequency of administration, and delivery routes are yet to be established. Moreover, exosome-based therapies may elicit unforeseen immunogenicity, especially when derived from allogeneic sources, necessitating rigorous safety evaluations. Additionally, the scale-up of exosome manufacturing under GMP-compliant conditions is still technically and economically challenging. Addressing these translational barriers is crucial for transforming exosome-based therapies from experimental platforms into viable clinical interventions. The integration of exosomes with biomaterial scaffolds and physical stimuli will offer a promising avenue for developing effective, multi-faceted treatments for OA. Furthermore, designing “smart” exosomes with targeted delivery capabilities or artificial intelligence (AI)-engineered cargos may enhance therapeutic efficacy and specificity. Ultimately, exosome research in OA stands at a promising yet formative stage. By addressing fundamental scientific questions and overcoming technical and regulatory barriers, future investigations can unlock the full therapeutic potential of exosomes for immune modulation and tissue regeneration in OA.
